# miRNAs and Melanoma: How Are They Connected?

**DOI:** 10.1155/2012/528345

**Published:** 2011-08-10

**Authors:** Adriana Taveira da Cruz, Miriam Galvonas Jasiulionis

**Affiliations:** ^1^Microbiology, Immunology and Parasitology Department, Universidade Federal de São Paulo, 04039-032 São Paulo, SP, Brazil; ^2^Pharmacology Department, Universidade Federal de São Paulo, 04039-032 São Paulo, SP, Brazil

## Abstract

miRNAs are non-coding RNAs that bind to mRNA targets and disturb their stability and/or translation, thus acting in gene posttranscriptional regulation. It is predicted that over 30% of mRNAs are regulated by miRNAs. Therefore these molecules are considered essential in the processing of many biological responses, such as cell proliferation, apoptosis, and stress responsiveness. As miRNAs participate of virtually all cellular pathways, their deregulation is critical to cancer development. Consequently, loss or gain of miRNAs function may contribute to tumor progression. Little is known about the regulation of miRNAs and understanding the events that lead to changes in their expression may provide new perspectives for cancer treatment. Among distinct types of cancer, melanoma has special implications. It is characterized as a complex disease, originated from a malignant transformation of melanocytes. Despite being rare, its metastatic form is usually incurable, which makes melanoma the major death cause of all skin cancers. Some molecular pathways are frequently disrupted in melanoma, and miRNAs probably have a decisive role on these alterations. Therefore, this review aims to discuss new findings about miRNAs in melanoma fields, underlying epigenetic processes, and also to argue possibilities of using miRNAs in melanoma diagnosis and therapy.

## 1. Introduction

Gene expression profiles characterize cells of specific tissues. Alterations on these patterns can promote cell homeostasis disruption leading to the appearance of some diseases, including cancer. In this regard, it is very important to comprehend how gene expression is regulated. One of the mechanisms of gene control is associated with the dynamic equilibrium between mRNA translation and its degradation and this process is intermediated by a special class of noncoding small RNAs. miRNAs (microRNAs), siRNAs (small interfering RNAs), and piRNAs (Piwi-interacting RNAs) are some elements that characterized the group of noncoding small RNAs, and the main differences between them are their molecular origin, biogenesis course, and size (for review see [1, 2]). These tiny molecules participate directly in gene expression outcome by physical interaction with mRNAs [3] and indirectly through aiding heterochromatin formation [4]. Therefore, due to their ability in interfering in transcriptome, small RNAs virtually participate on all biological processes. piRNAs and siRNAs seem to be important in gametogenesis and retrotransposon silencing of mammalian germ line [5, 6], as well as embryo development. Recently, it was demonstrated that some changes on small RNA expression pattern occur during mouse embryo development. These alterations encompass reduction of piRNAs and siRNAs expression with a simultaneity increase of miRNA expression. As a consequence, in somatic mammalian cells it is observed a predominance of miRNA expression compared to other small RNAs [7]. In fact, miRNAs are one of the most well-characterized small RNAs, although much about them still remains unclear. The first miRNA was discovered by Lee and colleagues in *Caenorhabditis elegans*. They demonstrated that miRNA *lin-4* negatively regulates LIN-14 protein expression and it is indispensable for normal progress of postembryonic developmental events in this worm [8]. Since that, miRNAs were found in a variety of organisms. Collective studies with multiple species demonstrated that some of these tiny molecules are extremely conserved through evolution [9]. The total amount of miRNAs described has also increased. Until now, it was identified more than 600 and 450 miRNAs in humans and mice, respectively, (http://www.microRNA.org/). These numbers tend to increase as a result of technology advances, such as high-throughput sequencing [10]. For these reasons, miRNAs were classified as a major class of gene regulatory molecules with expectancy of over 30% of human coding genes been directly regulated by them [11].

miRNAs expression considerably change on tumor cells; some miRNAs that negatively regulates oncoproteins are downregulated during malignant transformation cells while others that target mRNA of tumor suppressors are upregulated. These miRNAs are known as tumor suppressor miRNA and onocogenic miRNA (oncomiRs), respectively, [12].

Considering all different types of malignant tumors, melanoma has special implications. Although this malady is rare compared to other skin tumors, it depicts a large socioeconomic impact, especially for the fact that it has an elevated incidence among young people [13]. Melanoma develops from melanocyte malignant transformation, which is responsible for melanin synthesis. This pigment is distributed among epidermal keratinocytes, in order to avoid possible DNA lesions promoted mainly by ultraviolet (UV) radiation [14]. Once melanocytes are often under substantial genotoxic stress conditions [15], these cells are considerably more resistant to apoptosis [16, 17]. This could explain the absence of efficient treatments for metastatic melanoma [18, 19]. As a result, metastatic melanoma victims show high mortality rates with survival average around 6 months. Hence, melanoma treatments are basically limited to tumor surgical excision when early diagnosed [20]. 

Molecular changes involved in melanoma initiation and progression are caused by genetic and epigenetic alterations. BRAF activation mutation and Cyclin D1 gene amplification are well-characterized genetic alterations while global DNA hypomethylation and modifications in histone marks correspond to epigenetic changes, both frequently observed in melanoma tumors [21].

In this regard, the aim of this paper is to review the field involving melanoma, miRNAs, and epigenetics and to access the influence of these molecular findings in melanoma therapy perspective. Additionally, it will be discussed the alterations on the components of miRNAs biogenesis machinery during cancer development.

## 2. Disruption of miRNA Biogenesis Pathway and Its Implication in Tumorigenesis

miRNAs are synthesized in a sequential way comprising two principal events that involve Drosha and Dicer RNAses III enzymes [22]. Subsequently, mature miRNAs are loaded in a singular complex named RISC (RNA-induced silencing complex), where mRNA translational repression occurs [23]. In the past, scientist believed that miRNAs were only transcribed by RNA polymerase II, however now it is also showed that some are transcribed by RNA polymerase III [24]. In mammalian, miRNAs coding sequences are mainly distributed in tandem through the genome and these miRNA clusters are preferentially transcribed as polycistronic transcribed unit [1]. Besides, gene mapping reveled that many miRNA sequences are located in cancer-associated genome regions (CAGRs) [25], and this could explain the frequent modifications in miRNA expression during tumor progression. Indeed, global miRNA expression is frequently downregulated in cancer cells [26, 27].

Primary miRNAs (pri-miRNA) are the first precursors of miRNAs. They are formed by several kilobases long and stem-loop structures. Like other mRNAs, pri-miRNAs undergo posttranscriptional changes, such as 5′-cap and 3′-poly(A) tail [28]. pri-miRNAs are processed in the nucleus by Drosha, in the presence of DGCR8 (DiGeorge syndrome critical region) cofactor. Wang and collaborators demonstrated that mouse stem cells *Dgcr8 *gene knockout presented alterations in miRNA synthesis, which resulted in proliferation and differentiation defects [29]. Therefore, alterations in Drosha and DGCR8 expression can indirectly modify gene profile. In fact, it was observed that Drosha deregulation can contribute to malignant progression. Muralidhar and colleagues showed that Drosha transcript increases during the progression of cervical squamous cells carcinoma (SCC) and such rise is accompanied by miRNA profile changes [30]. Drosha augment was also observed in oesophageal SCC and it was correlated with poor prognosis [31]. Although modifications in Drosha and DGCR8 expression have been reported in some tumors, there is no information about the expression of these enzymes in melanoma. Therefore, understanding the first steps of miRNA processing pathway along melanoma progression may provide important insights about this cancer.

Drosha acts on pri-miRNA resulting in pre-miRNAs and leading a 5′ phosphate and a 2-nucleotide 3′ overhang at the hairpin base. Pre-miRNAs have 60–70 nucleotides (nt) and are exported from nucleus to cytoplasm through RAN GTPase Expotin-5 (XPO5) [32]. Pre-miRNA nuclear translocation is tightly regulated in normal cells. Melo and colleagues [27] demonstrated that *XPO5* mutations lead to nuclear pre-miRNA accumulation. Such alteration was responsible for cell homeostasis deregulation and contributed to tumor progression [27]. In fact, *XPO5 *deletion was observed in different malignances [33].

On cytoplasm, pre-miRNAs are further processed by Dicer into miRNAs duplexes of approximately 22 nt long. Dicer probably recognizes 2-nucleotide 3′ overhang at the base of stem loop [34]. As signature of RNAse III protein family, Dicer also leads 5′ phosphates and 2-nucleotide 3′ overhangs in the mature miRNA [35]. Dicer enzyme seems to be indispensable for miRNA biogenesis. While a group of miRNAs is processed independently of Drosha (i.e., mirtrons), there is no evidence for Dicer-independent miRNA processing [36]. Although Dicer is an ubiquitous protein, its expression regulation may control miRNA synthesis in specific contexts. Actually, it was verified that Dicer participates on melanocyte differentiation but such involvement was not noted in other cell types [37]. During melanocyte differentiation, miRNA expression increases while pre-miRNA expression is maintained constant. Dicer expression rises simultaneously, showing that miRNA pattern is also a consequence of posttranscriptional alterations and not only a result of the transcription itself. Analyses of specific miRNAs showed that miRNA-17 expression is directly controlled by Dicer and this miRNA has great influence in apoptosis pathway. In this way, Dicer disruption can contribute with tumor progression since it also alters expression of specific miRNA, related to tumorigenesis [37]. In a pilot study, it was verified that Dicer expression is higher in cutaneous malignant melanomas (CMM) compared to benign melanocytic nevi (BMN). Besides, Dicer expression in CMM is significantly correlated with Breslow tumor thickness [38].

Dicer activity can be regulated by some proteins, such as TRBP (Tar RNA binding protein) [39], PACT (PKR activator) [40], and KSRP (KH-type splicing regulatory protein) [41]. For example, TRBP and Dicer association is required for efficient RNA silencing mediated by siRNAs and miRNAs [39]. Some colorectal and endometrial cancer cells with microsatellite instability presented inactivating mutations of *TRBP2 *gene [42]. As consequence of such inactivation, TRBP2 protein expression was reduced and miRNA processing was disturbed. miRNA biogenesis disorder was promoted by DICER1 destabilization and reintroduction of wild-type TRBP2 inhibited tumor growth and restored miRNA normal synthesis. Analysis of *TRBP2 *gene disruption in human primary tumors of nonpolyposis colon cancer and sporadic colon cancer revealed that this alteration is frequent, showing the importance of that protein to maintain cell homeostasis [42]. In reality, TRBP is considered a potential oncogene. Its expression is linked to increased cell proliferation rates, and its overexpression is associated with malignant phenotype [43]. Therefore, TRPB has an important role in tumorigenesis processes once its alteration can disrupt miRNA synthesis or lead to cell growth. 

Some pathways frequently disrupted in different tumor types also participate in the complex regulation of miRNA synthesis. Boominathan showed that members of p53 family, including p53, p63, and p73 may participate on miRNA biogenesis [44]. Therefore, miRNA deregulation in cancer can also be a consequence of p53 family components injury.

After Dicer cleavage, 22 nt RNA duplex is loaded onto Argonaute (Ago) protein to generate the effector complex RISC. One strand of this duplex, known as miRNA guide strand, remains linked with Ago while the other, named *miRNA, is degraded [1]. miRNA associates with mRNA by base complementarity [11], and such association can be total or partial, resulting in mRNA destabilization or degradation [3]. Normally, miRNAs bind in mRNAs by its 3′-untranslated region (UTR), but the binding through 5′-nontranslated or coding sequence of mRNAs also occurs [45]. Ago proteins are the principal components of RISC and they mediate repression of mRNA induced by miRNA targeting. Eight Ago proteins are encoded by human genome: Ago 1–4 and Piwi 1–4. Nevertheless, Ago2 is the only with cleavage activity [46]. Ago2 was also correlated with tumor development. Its expression is associated with the transformed phenotype in breast cancer cells [47]. The data discussed above were summarized in Table 1.

## 3. miRNAs Associated with Pathways Involved with Melanoma Development

As discussed above, miRNA profile is different in cancer compared to normal cells. Therefore, many authors have focused their studies on the mechanism in which such variation may contribute to tumor progression. In this way, melanoma research involving miRNAs have been developed. New findings on mRNA targets and pathways specifically controlled by miRNAs will improve the comprehension of this malignancy, the identification of new biomarkers, and the development of novel therapies. Some well-characterized disrupted cellular processes have been associated with melanocyte malignant transformation. BRAF and p16^INK4^ mutations, E-cadherin loss, and increased telomerase activity are frequently observed in melanoma cells [48]. Moreover, several molecular alterations are connected with particular stages of this cancer progression [49].

In human skin microenvironment, melanocytes and keratinocytes express E-cadherin while fibroblasts and endothelial cells express N-cadherin. E-cadherin is the major adhesion molecule that mediates association between melanocytes and keratinocytes [50]. This connection permits keratinocytes to control important paths of melanocytes, like differentiation, proliferation, and expression of cell surface receptors. During initial phases of melanoma progression, changes on cadherin expression pattern are frequently observed. Firstly, E-cadherin expression becomes heterogeneous, then gets diffused distributed on cytoplasm of dysplastic nevus and finally are lost in melanoma [51]. Through this process, melanoma cells also express N-cadherin and acquire the ability to associate with fibroblasts and endothelial cells [52]. The loss of E-cadherin is a critical event once melanocytes that do not express this molecule are not regulated by keratinocytes [50]. Cadherin switch probably leads to subsequent gene expression changes and foments tumor cell invasion and migration. Therefore, melanocytes that do not survive on dermis, after this switch would be able to live in that new ambient [51]. 

Despite the switch of E-cadherin to N-cadherin being a critical event on melanoma development, it was demonstrated that other adhesion molecules participate in this tumor progression. Mueller and Bosserhoff [53] showed that cadherin-11 expression is also lost in melanoma cells. HOX-C8 promotes reduction of cadherin-11 expression contributing to its regulation [53]. HOX family proteins are important during embryogenesis, but also play a role in adult eukaryotic organisms. In adults, these proteins participate in the control of cell growth and differentiation and cell-cell and cell-matrix interactions [54]. Deregulation of cadherin-11 observed in melanoma is an indirect consequence of decrease in miR-196a expression [53]. miR-196a directly regulates the expression of transcript factor HOX-C8 through linking on its 3′UTR mRNA region [55]. Disrupted *HOX *gene expression correlates with transition from differentiated to an undifferentiated cell type state [53]. This phenotype modification is tightly related with malignant transformation and metastatic potential [26, 53]. Mueller and Bosserhoff [53] also showed that other adhesion proteins controlled by HOX-C8 such as osteopontin and calponin-1 were also deregulated in melanoma. *In vitro *investigation of HOX-C8 alteration mediated by miR-196a showed that miR-196a reexpression in melanoma cells reduced their invasive capacity and *in vivo *examination demonstrated that injecting these cells over-expressing miR-196a prevented tumor growth in 50% of mice analyzed [53]. This work reinforces the importance of regulating components as miRNAs to avoid cell homeostasis disruption and malignant transformation. Moreover, this work suggests miR-196a as a target for new therapies against metastatic melanoma. 

Changes in cadherin profile are also associated with epithelial to mesenchymal transition (EMT). EMT is a physiologic process related to cell migration and invasion during embryonic development. Nevertheless, it is also linked with tumor progression and metastases [56]. In melanoma, the transcript factor SNAIL1 induces EMT by repressing E-cadherin transcription and inducing N-cadherin [48]. miR-200 family is also associated with EMT control, through Zeb transcriptional factors suppression, which are in turn other repressors of E-cadherin [57]. miR-200 components also promote E-cadherin-dependent junction formation and inhibit cell migration [58]. In some cancers, miR-200 family expression is downregulated and contributes to EMT and cell invasion [59]. However, it was verified that the expression of some members of miR-200 family is increased in melanoma cells [60–62]. Elson-Schwab and colleagues [63] demonstrated that increased expression of miR-200 in melanoma does not abrogate invasion of these cells, instead it promotes a switch between invasive pathways. Indeed, superexpression of miR-200a or miR-200c can increase the invasive capacity in some melanoma cell lines. *In vivo, *melanoma cell invasion is influenced by cell morphology and by changes on modes of invasion. *In vitro, *experiments showed that miR-200a is related preferentially with elongated manner (mesenchymal-type) while miR-200c conducts to rounded mode of invasion (amoeboyd-like) [63]. These findings corroborate the specific role of miRNAs in a determined context. They suggest miR-200 regulation and activity are circumstance dependent and may favor cancer progression in some tumor types. 

MITF (microphthalmia-associated transcription factor) protein is an important component of melanocyte development. This molecule induces the expression of transcription factors related with melanocyte differentiation, such as the Myc family member bHLH-Zip (Basic helix-loop-helix leucine zipper) [64]. bHLH-Zip is responsible for encoding other melanocyte determinants as tyrosinase (Tyr), tyrosinase-related protein-1 (Tyrp-1), and DCT/Tyrp-2 [65]. Besides its role in melanocyte differentiation, MITF expression is also related to melanocyte survival, proliferation, and cell cycle progression [66]. Moreover, MITF continued expression seems to be essential to melanoma proliferation and survival. Actually, it was proposed that MITF acts as lineage survival oncogene in melanoma [67]. Generally, melanoma cells express high levels of MITF, but this condition differs greatly among melanoma cell lines and cells in tumor tissue [66]. In fact, since MITF reduces melanoma cell proliferation and tumorigenicity, there is less MITF expressed in melanoma cells than in normal melanocytes [68]. These data suggest a tight control of this protein. *Mitf *3′UTR mRNA sequence probably has a special role on its own regulation. Human and mouse *Mitf *mRNA have a relatively large 3′UTR sequence compared to 3′UTR of other mRNAs and its 3′UTR region shows high indices of conservation among different vertebrates, a special characteristic since UTR sequence are not translated [69]. As MITF is determinant in cancer progression, its regulation by miRNA seems to be especially important. Haflidadóttir and coworkers [69] investigated predicted miRNAs responsible to target *Mitf* mRNA 3′UTR sequence and the functionality of these miRNAs in Mitf expression in melanoma cells [69]. These authors demonstrated that miR-148 binds in *Mitf *3′UTR mRNA and downregulates its expression. miR-137 is another miRNA that directly links in *MITF* 3′UTR mRNA and controls its expression, and it was previously reported by Bemis and coworkers [70]. Hence, mutation or loss of *Mitf *3′UTR sequence is likely to occur during melanoma progression. In addition, miR-148 and miR-137 expression changes are critical to melanoma. Indeed, miR-148 expression is diminished in melanoma lineages [60]. Moreover, in some melanoma cells it was noted a variable nucleotide tandem repeat (VNTR) in the pri-miRNA-137 that alters the function of mature miR-137 and hinders this miRNA to promote MITF repression [70]. miR-182 also directly regulates MITF and FOXO3 expression [68]. This miRNA is located in a DNA region frequently amplified in melanoma cells, flanked by *c-Met and BRAF *oncogenes and was found upregulated in melanoma cell lines. Downregulation of miR-182 represses melanoma cell invasiveness and promotes cell death while its ectopic expression enhances metastatic potential *in vivo *[68]. Therefore, it has been demonstrated that some miRNAs are involved with MITF expression in melanoma. Furthermore, MITF probably has an important role in miRNA expression control. Mazar and collaborators [73] showed that miR-211 expression is reduced in melanoma cells related to melanocytes from human tissues and from cell lines, and that this alteration contributes with the malignant metastatic phenotype [71]. They also verified that miR-211 directly regulates the expression of KCNMA1 protein, which in turn is upregulated in melanoma cells. KCNMA1, that was already linked with other tumor types [71], is associated with high proliferation rates and invasion capacity. Moreover, it was suggested that miR-211 perhaps is under control of MITF. The transcript sequence for this miRNA is located in the sixth intron of *TRPM1* gene, *locus* that encodes genes that was previously suggested as melanoma aggressiveness suppressors. *TRPM1* gene expression is controlled by MITF through its association with *TRPM1* promoter [72]. Mazar and collaborators [73] obtained evidences that miR-211 expression is regulated by MITF via TRPM1 promoter. These events generated one hypothesis for the mechanism in which MITF can suppress melanoma metastasis [73]. In conclusion with this findings, if MITF is reduced in melanoma cells, it will have less expression of TRPM1 and consequently of miR-211. Downregulation of miR-211 leads to increased KCNMA1 protein level, favoring the maintenance of malignant phenotype. 

Other miRNAs were described as critical molecules in melanoma development due to their participation in the control of cell cycle components. Downexpression of miRNA-193b was verified in metastatic melanoma cell lines compared to benign nevi and this reduction was correlated with increased cell proliferation. Further investigations demonstrated that this unbalance was a consequence of enhanced CYCLIN D1 expression [74]. Moreover, functional experiments showed that this regulation is a consequence of direct binding of miR-193b to 3′UTR mRNA of *CYCLIN D1*. 

Like *Mitf*, *CYCLIN D1 *has more than one miRNA validated as promoting its repression in melanoma cells. The validation refers to the physical interaction of miRNA and mRNA target, and its cellular outcomes. It is already known that single miRNA has diverse mRNAs targets and that specific mRNAs can be targeted by several miRNAs. Nevertheless in melanoma area, only few miRNAs were confirmed as establishing such interaction. In fact, some works showed a global pattern of miRNA expression in melanocytes versus primary melanoma or versus metastatic melanoma in order to determine a specific miRNA signature for this cancer [60, 70, 75]. However, these works represent only the first step in melanoma miRNA profile characterization, once much more still need to be done. Only small number of miRNAs and their respective mRNA target were validated in melanocytes/melanoma. In this way, besides miR-193b, miR-let7b was also prompted as targeting *CYCLIN D1 *[61]. miR-let-7b has a particular importance in cancer progression because it regulates RAS oncogenes [76] and is also involved in stem cell maintenance [77]. Global analyses of some miRNAs in melanocytes compared to primary melanoma human tissue showed that all members of miR-let7 family were downregulated. Luciferase assays demonstrated that miR-let7b binds to 3′UTR *CYCLIN D1* mRNA and its superexpression is coupled with small proliferation rates of melanoma tumor cells and with reduction of anchorage-independent tumor growth [61].

Genetic alterations of pathways that involve this cell cycle component and in this gene itself are frequently observed in melanoma. CDNK2A *locus *is commonly altered, both in sporadic and familial melanomas. From that sequence, it is transcribed two important proteins: p16^INK4a^ and p14^ARF^. In a simplified way, p16^INK4a^  is responsible for CYCLIN D1/CDK4 complex destabilization. CDK4 activation mediated by CYCLIN D1 is one of the main signals promoting cell cycle progression through G1 to S phase, because it prompts Rb to liberate E2F transcription factor. E2F without Rb stimulates transcription of many genes related with proliferation. Therefore, mutation in *p*16^INK4a^  can contribute to cell uncontrolled proliferation. In the order hand, p14^ARF^  interacts with MDM2 and blocks MDM2 to target p53 to degradation, contributing with p53 stabilization. So, *p*14^ARF^ mutations can support deregulation in cell apoptosis mediated by p53, favoring cell survival. 

Unlike many cancers, *p53* is rarely mutated in melanoma. However, the equilibrium of its function can be changed by other factors, as exemplified by p14^ARF^ alterations. Increase of CYCLIN D1 and CDK4 proteins in melanoma cells can also be a consequence of gene amplification. Nevertheless, it is estimated that this only happens in 4% of melanomas. In a study of Cyclin D1 role in melanoma progression it was showed that a considerable number of tumors had its expression raised independently of copy number amplification [78]. Thus, what more could be responsible for CYCLIN D1/CDK4 increase in melanoma cells? miRNAs could be a reasonable answer, as showed the new discovers underlying miR193b and miR-let7b. 

Moreover, it was demonstrated that miR-205 is also involved in the regulation of cell cycle components. E2F1 is a member of E2F family responsible for transactivation of genes involved with chromosomal DNA replication and cell cycle progression. It was showed that miR-205 binds at 3′UTR *E2F1* mRNA, promoting its repression. Indeed, E2F1 is over-expressed while miR-205 is significantly suppressed in melanoma cells related to normal human melanocytes. The result of that is the high proliferation rate of melanoma cells. Indeed, proliferative capacity of melanoma cells is suppressed when miR-205 was super-expressed [79].

Despite its possible role in cell cycle regulation, miR-let7b also participates in tumor invasion mechanisms in melanoma. Metalloproteinases (MMPs) are proteins that promote extracellular matrix (ECM) components degradation. Such phenomenon is strictly correlated with cell invasive capacity and high expression of these enzymes is frequently observed in tumors. Basing (Bsg) is other protein involved with ECM degradation, because it stimulates MMP-1, -2, -3, and -9 syntheses. Bsg level is also increased in tumor cell and, in this case, it provokes adjacent fibroblasts and tumor cells to produce MMPs [80]. miR-le7b indirectly downregulates MMP9 through its association with Bsg in mouse melanoma cell line [81]. The consequence of the miR-let7b repression in melanoma cell line was the same as showen in the studies already reported about CYCLIN D1 and the same miRNA, that is, proliferation and colony formation reduction. Yet, it was showen decreased invasion and migration *in vivo* and inhibition of metastases in a mouse model [81].

These are some studies that indicate the importance of miRNAs role, regulating key components of the tumorigenic process in melanoma (recapitulated on Table 2). Besides these miRNAs, there are others already described. However, much about them still have to be clarified.

## 4. Epigenetic Mechanism, miRNAs, and Melanoma

Although whole human genome has already been sequenced, our comprehension about the intricate network of gene expression is far from being deciphered. One reason for this is that the information necessary for gene transcription is not restricted at the linear sequence of DNA nucleotides. The mechanisms underlying gene expression control are complex and involve methyl radical associated with DNA bases and histones tail marks. Interchange between heterochromatin and euchromatin states promotes alteration in DNA structure and modify chromatin accessibility to transcription factors. Epigenetic field encompasses these findings about gene expression control. DNA methylation and posttranslation modifications in histones are the principal mechanisms in which genes expressions are altered without any changes in nucleotides sequence. 

DNA methylation promotes protection for organism, limiting the expression of foreign DNA elements and endogenous transposons [82] and also regulates expression of genes involved with differentiation [83]. DNA methylation process corresponds to the addiction of methyl group in DNA bases. In mammalian, such processes occur preferentially on cytosine residues in the context of CpG dinucleotides [84]. These dinucleotides are normally distributed in tandem, forming CpG islands that preferentially takes place in repetitive sequences and gene promoters [85]. DNA methyltransferase (DNMTs) enzymes are responsible for catalyzing the addiction of methyl group on CpG dinucleotides and for that reason they are in charge of DNA methylation pattern [86]. Among DNMTs already described, DNMT1, DNMT3a, and DNMT3b are the only that have catalytic activity, being determinant in DNA methylation process [87]. Despite their cooperative role; it is showed that DNMT3a and DNMT3b are involved in the establishment of new DNA methylation profile (*de novo *methylases) while DNMT1 maintains that profile during cell division. Though, DNMT3a and DNMT3b have special function during early development and gametogenesis whereas DNMT1 has important meaning in homeostasis of somatic cells. In fact, inactivation of Dnmt3b results in embryonic lethality, Dnmt3a knockout mice die shortly after birth [88] and more, mutations in the human *DNMT3b* gene are connected with ICF (Immunodeficiency, Centromere instability and Facial anomalies) syndrome [89].

Tumor cells have different DNA methylation pattern compared to nontransformed cells. In cancer is observed a global DNA hypomethylation and specific hypermethylation linked with tumor suppressor gene promoters. Deregulation of DNMTs is probably involved with this unbalance. In lung squamous cells carcinoma, DNMT1 super-expression is involved with poor prognosis and over-expression of DNMT1 and DNMT3b is correlated with hypermethylation of tumor suppressor genes [90]. Some miRNAs were described targeting *DNMTs *mRNA. These miRNAs were also associated with DNA methylation changes and tumorigenesis development. Braconi and colleagues verified that DNMT1 expression was elevated in some cholangiocarcinoma cell lines compared to nonmalignant human cholangiocyte lineage, and that increased was an indirect consequence of interleukin-6 (IL-6) over-expression [91]. Inflammation associated with IL-6 was correlated with this tumor type through autocrine and paracrine mechanisms, promoting increase of proliferation rates and cell survival [92]. In an attempt to determine molecular pathways in which IL-6 regulates DNMT1, the authors searched for miRNAs that concomitantly were reduced in cholangiocarcinoma cell lines and that had nucleotide sequence complementary to 3′UTR mRNA region of *DNMT1*. Indeed, they showed that some miRNAs fulfilled these conditions but only miR-148a and miR-152 were capable to bind on mRNA 3′UTR region of *DNMT1* and reduced luciferase activity. For determining the implication of these findings, they also analyzed the effect of enforced expression of miR-148a and miR-152 in cholangiocarcinoma cell lines over p16^INK4a^ and Rassf1 proteins. These tumor suppressors, that do not have complementary sequence to these miRNAs and that were hypermethylated and underexpressed in these cell lines, have their expression increased in the presence of miR-148a or miR-152 [91]. Therefore, from these works it can be emphasized that alterations of few miRNAs are able to promote deregulation of a huge group of genes, at least for genes that have CpG island in their promoter and are under DNMT1 regulation. These findings also show that environment perturbation, such enhance of IL-6 production, can modify miRNA expression. Furthermore, these studies suggest miRNAs as modulators of DNA methylation, through DNMT1 control. Despite these observations have been made in cholangiocarcinomas, they also may have some implication in melanoma development. In a murine model of melanocyte malignant transformation, Molognoni and coworkers showed that Dnmt1 expression increased through different stages of melanoma progression, getting it maximum expression in malignant metastatic cells [26]. Moreover, other authors have already described that miR148a are diminished in human metastatic melanoma lineages [60]. Taking these data together, there is evidences that similar regulation may occur in melanoma tumors. However, this hypothesis must be tested before being generalized because like it was also discussed, equal miRNAs can have different implications depending on tumor type.

Duursma and coworkers verified that miR-148 can also regulate the expression of some DNMT3b variants [93]. Unlike what is often observed, miR-148 is not linked to 3′UTR mRNA *DNMT3b *region, but instead is associated to protein coding sequence (CDS). This site corresponds to a conserved evolutionary sequence present in the *DNMT3b* splice variants *DNMT3b1*, *DNMT3b2*, and *DNMT3b4*, but not in *DNMT3b3. *Other miRNAs were also described participating in DNMTs regulation. miR-29 family members (miR-29a, -29b, and -29c), which are inversely expressed related to *DNM3a* and *DNMT3b*, in lung cancer tissue are directly associated with 3′UTR mRNA region of *DNMT3a* and *DNMT3b *[94]. miR-29s alteration in lung tumor also contributed to changes in DNA methylation profile. Enforced expression of miR-29s in these tumor cell lines restored normal DNA methylation pattern. In other words, suppressor genes that were silenced in lung cancer by methylation process were reexpressed in the presence of miR-29s.

As showed, miRNAs can regulate DNMT expression and indirectly modify DNA methylation patterns. Nevertheless, it seems that miRNA expression can also be controlled by DNA methylation. To further address this question, Han and colleagues compared miRNA expression profile in colorectal carcinoma, cell line HCT116 double knockout for DNMT1 and DNMT3b with its parental control [95]. Double knockout cells had 95% of genomic DNA methylation reduction related to parental HCT116 cells and miRNAs presenting significant increase in their expression. Therefore, its result supports the proposition that miRNAs can be controlled by methylation process, even in an indirect way. In fact, other works elucidated the importance of DNA methylation in the regulation of miRNA expression and its relation with tumor development, including in melanoma. In one, the authors showed that miR-34a expression can be epigenetically repressed [96]. Firstly, it was demonstrated that tumor suppressor p53 modulates miR-34a expression by binding in its consensus region presented at miR-34a locus [97]. mir-34a expression displays reduced in many tumor cell lines, including prostate, pancreas and breast carcinoma and also in melanoma cell lines [96]. Ectopic expression of miR-34a in primary and cancer cells promotes apoptosis or cell cycle arrest, coping the phenotype mediated by p53 expression [97]. These results highlighted the importance of miR-34a in cancer development and also emphasize the role of p53 in the regulation of that miRNA expression. Nevertheless, Lodygin and colleagues, analysing the genomic region upstream of the p53 binding site in the *miR-34a *gene, found a prominent CpG island in that locus [96]. Furthermore, studying the effect of *miR-34a *island, they demonstrated that miR-34a expression is regulated by DNA methylation. Importantly, the silencing of *miR-*34a mediated by DNA methylation was dominant over its transactivation by p53 after DNA damage. In that work, analyses of miR-34a CpG methylation revealed that 43.2% and 62.5% of melanoma cell lines and primary melanoma samples, respectively, were methylated. Repression of miR-34a by methylation can provide a selective advantage for cancer cells that have lost p53 function [96]. So, those findings show how critical is the role of epigenetics under miRNAs control in melanoma, and other cancers, development. 

Methylation of the human *miR-let-7a-3 *gene also seems to be involved with tumorigenesis. Brueckner and coworkers demonstrated that *miR-let-7a-3* gene is associated with well-defined CpG island [98]. Moreover, these authors verified that miR-let-7a-3 methylation is prevalent in lung human normal tissues and is reduced in lung adenocarcinoma. In that work, it was also established that DNMT1 and DNMt3b cooperatively methylate *miR-let-7a-3 *CpG island. As miR-let-7a-3 is part of the archetypal miR-let-7 gene family, it may have similar targets. In fact, the authors showed that miR-let-7a-3 probably regulates mRNAs that also are controlled by miR-let 7 family members. As already discussed here, miR-let-7 family has a special role in melanoma, suggesting miR-let-7a-3 can also be a candidate in melanoma tumorigenesis process [98]. The information about miRNAs and epigenetic machinery discussed in this section were compiled in Table 3. 

## 5. Therapy: General Panorama X Perspective

Through this paper, the importance of miRNAs in tumor development, especially in melanoma, was highlighted. Disruption of key components of miRNA biogenesis and their involvement with tumorigenesis was further explored. However one significant question surrounding miRNAs and melanoma still needs to be clarified: how these findings about miRNAs can help diagnoses and treatment of melanomas? Recently, Vidwans and colleagues proposed a molecular model for progression of melanoma disease and more, suggested different types of treatments for distinct molecular lesions [99]. In that work, miRNAs were not included as molecular lesion and were not used to classify melanoma tumors probably, not because they are not critical in the process of melanocyte malignant transformation, but for the reason that the knowledge concerning miRNAs still needs to be further accessed. Many discoveries made about miRNAs and their role in melanoma are only in the beginning. Vidwans and collaborators [99] that suggested an alternative classification to melanoma against the standard approach based mainly in tumor morphology (Clark) or thickness (Breslow), supported their proposition in well-characterized molecular alterations underlying this tumor type, and in that case miRNAs could not be included. Therefore miRNAs are strong candidates to be shared in melanoma classification by Vidwans and coworkers; however they require to be more investigated. Therefore studying miRNAs will improve new proposes of melanoma therapy. Despite that, some initial works have already been made, targeting miRNAs, melanoma, and therapies and searching for miRNA biomarkers.

Biomarkers are molecules that permit the detection of cancer in early stages of development, providing the following of tumor progression or regression and supplying the supervision of treatment efficacy [100]. Many evidences made miRNAs as powerful biomarker candidates: miRNA pattern expression seems to be singular for different types of cancer, their expression in body fluids, including blood, can be easily detected by PCR-related assays, and, compared to other biomarkers, miRNAs are remarkably stable molecules [101], being capable to be identified even in formalin-fixed paraffin-embedded tissue. Moreover, circulating miRNAs can represent a non-invasive diagnostic biomarkers for distinct types of cancer [102]. In this way, some researchers focus their work in the potential of miRNAs as biomarkers. Holst and colleagues [103] looked for miRNAs that would improve the classification among a typical nevi (AN) from common acquired nevi (CN). AN is defined as elevated melanocyte lesion, with more than 5 mm in diameter and its presence is considered an independent predictor of malignant melanoma. These lesions can be characterized by their morphological appearance: irregular or asymmetric outline, variable pigmentation, and indistinct boarders. However AN is not histologically well defined, making differentiation between AN and CN diffcult [103]. Therefore, from a study with 41 patients, 19 with CN, and 22 with AN, Holst and collaborators [103] indentified 36 potential miRNAs that could be used to discriminate CN from AN. Moreover, in hierarchical cluster analysis of these miRNAs, AN was clusterized into two groups, reinforcing the fact that these kind of lesions are molecularly heterogeneous [104]. Quantification by real-time RT-PCR of picked miRNAs showed that miR-125 was downregulated and miR-let-7c was upregulated in AN compared to CN [103]. So these miRNAs can be used to distingue CN from AN. miR-125b has already been proposed for that same group as prognostic marker of metastatic melanoma [105]. They demonstrated that miR-125 was under-expression in malignant melanoma capable to generate lymph node micrometastases related to melanoma unable to metastasize. In addition, it was proposed that miR-125b alteration in early cutaneous malignant melanoma may indicate augmented probability of this tumor to become metastatic.

Other miRNA proposed as useful for melanoma diagnostic was miR-221. It was reported that miR-221 expression increases during different steps of melanocyte malignant transformation and also that its expression is almost unnoted in normal human melanocytes [106]. Kanemaru and coworkers investigated miR-221 levels in serum from different melanoma patients [107]. They found that miR-221 expression from malignant melanoma patients was significantly higher compared to the normal individuals, and also that, patients with melanoma in stage I–IV have miR-221 expression more elevated related to patients with *in situ *melanoma. Therefore, the quantification of miR-221 in serum could be used as non-invasive examination to differentiate *in situ* melanoma from the others. Moreover, these authors also observed that miR-221 expression was correlated with tumor thickness, suggesting that there is a direct association among miR-221 and prognostic. miR-221 levels as well decreased after tumor surgical removal and increased in patients with melanoma recurrence. Although miR-221 expression increases are also noted in other tumors [102, 108, 109], serum expression of that miRNA linked with clinical findings can be promising in melanoma detection. 

Beside the fact that miRNAs can be utilized as biomarkers, these molecules can also influence melanoma therapy. One appropriate strategy to explore the potential of miRNAs in this field is developing drugs targeting oncogenic miRNAs. As already related in this paper, miR-182 is frequently over-expressed in melanoma. Huynh and colleagues showed that targeting miR-182 with anit-miR-182 promotes melanoma liver metastases reduction compared to control, in mouse model of melanoma liver metastasis [110]. Moreover, analyses of mRNA transcripts from tumors treated with anti-miR-182 showed that the expression of genes involving controlling survival, adhesion and migration augmented related to non-treated tumors. These authors suggest that administration of anti-miR-182 could be interesting therapeutic strategy for metastatic melanoma.

Relative to other molecules, such as cell cycle components or members of Ras/Raf/ERK pathway, few about miRNAs and melanoma was already described. Nevertheless, these small noncoding RNAs represent a great hope for understanding such aggressive disease. Therefore miRNAs might be faced as a new strategy for melanoma therapies development.

## Figures and Tables

**Table 1:** Alterations of miRNAs machinery biogenesis components and their role on tumorigenesis.

Altered components	Cell type	Consequence	Reference
*Dgcr8 *knockout	mouse stem cells	miRNA synthesis disruption, cell proliferation and differentiation changes	[29]
Drosha increase	squamous cells carcinoma	miRNA profile modification	[30]
*XPO5* mutation	in a subset of human tumors	pre-miRNA accumulation	[27]
Dicer increase	cutaneous malignant melanomas	—	[38]
*TRBP2 *mutation	colorectal and endometrial cancer cells	DICER1 destabilization and miRNA processing disturbed	[42]
Ago2 increase	breast cancer cells	more aggressive phenotype of breast cancer lineage negative for ER expression	[47]

**Table 2:** miRNAs expression changes related with melanoma progression and their targets.

miRNAs	mRNA target	Consequence	Reference
miR-196a decrease	*HOX-C8*	cadherin-11 expression modification	[55]
miR-200a or miR-200c super-expression	*—*	increase the invasive capacity	[63]
miR-148 diminished	*MITF*	contributes to malignant transformation	[69]
VNTR pri-miRNA-137 (miR-137)	*MITF*	contributes to malignant transformation	[70]
miR-182 gene locus amplification	*MITF *and* FOXO3 *	Increased of invasive and survival capacity and enhances metastatic potential	[68]
miR-211 decrease	*KCNMA1*	contribute with malignant metastatic phenotype	[71]
miRNA-193b decrease	*CYCLIN D1*	increased cell proliferation	[74]
miR-let7b decrease	*CYCLIN D1 *and* Bsg *	high proliferation rate	[61, 81]
miR-205 decrease	*E2F1*	high proliferation rate	[79]

**Table 3:** miRNAs involved with epigenetic machinery control and miRNAs controlled by components of epigenetic machinery.

miRNAs	mRNA target	Epigenetic mark	Reference
miR-148	*DNMT3B*	—	[93]
miR-29 family members	*DNMT3a* and *DNMT3b *	—	[94]
*miR-34a* gene	—	DNA methylation	[96]
*miR-let-7a-3* gene	—	DNA methylation	[98]
